# The biometric parameters of aniso-astigmatism and its risk factor in Chinese preschool children: the Nanjing eye study

**DOI:** 10.1186/s12886-021-01808-7

**Published:** 2021-02-03

**Authors:** Haohai Tong, Qingfeng Hao, Zijin Wang, Yue Wang, Rui Li, Xiaoyan Zhao, Qigang Sun, Xiaohan Zhang, Xuejuan Chen, Hui Zhu, Dan Huang, Hu Liu

**Affiliations:** 1 Department of Ophthalmology, The First Affiliated Hospital with Nanjing Medical University, 300 Guangzhou Road, Nanjing, 210029 China; 2 Department of Ophthalmology, Maternal and Child Healthcare Hospital of Yuhuatai District, Nanjing, China; 3 Department of Ophthalmology, Wuxi Children’s Hospital, Wuxi, China; 4 Department of Child Healthcare, The First Affiliated Hospital with Nanjing Medical University, Nanjing, China

**Keywords:** Aniso-astigmatism, Apgar score, Preschool children, Population-based study

## Abstract

**Backgrounds:**

Aniso-astigmatism may hinder normal visual development in preschool children. Knowing its prevalence, biometric parameters and risk factors is fundamental to children eye care. The purpose of this study was to determine the biometric components of aniso-astigmatism and associated maternal risk factors in Chinese preschool children.

**Methods:**

In the population-based, prospective cohort Nanjing Eye Study, children were measured for noncycloplegic refractive error using an autorefractor and for biometric parameters using an optical low-coherent reflectometry. The difference of total astigmatism (TA) between both eyes was calculated using cylinder power (non-vectorial aniso-TA was defined as ≥1.00 Dioptre Cylinder [DC] between both eyes) and by vector analysis (vectorial aniso-TA was defined as a difference of ≥0.5 in *J*_*0*_ or *J*_*45*_ between both eyes which is equivalent to 1.00 DC). The prevalence of aniso-TA was presented. Interocular biometric parameters were compared between with vs. without aniso-astigmatism group. In addition, risk factors were determined using multivariate logistic regression model.

**Results:**

Of 1131 children (66.90 ± 3.38 months, 53.31% male), the prevalence of non-vectorial aniso-TA was 1.95% (95% Confidence Interval (CI) = 1.14–2.75%), while the prevalence of vectorial aniso-TA was twice as common as non-vectorial aniso-TA, neither varying with sex or age. With aniso-TA eyes were more asymmetric in axial length and corneal curvature radius than without aniso-TA eyes. In multivariate logistic regression model, 5-min Apgar score less than 7 was significantly associated with higher risk of aniso-TA (vectorial aniso-TA: Odds Ratio (OR) = 6.42, 95%CI = 2.63–15.69, *P* < 0.001; non-vectorial aniso-TA: OR = 4.99, 95%CI = 1.41–17.68, *P* = 0.01). Being twin or triple was significantly associated with higher risk of vectorial aniso-CA (OR = 2.43, 95%CI = 1.05–5.60, *P* = 0.04). Pre-term delivery (OR = 2.60, 95%CI = 1.09–6.15, *P* = 0.03) and post-term delivery (OR = 3.61, 95%CI = 1.31–9.96, *P* = 0.01) were significantly associated with higher risk of vectorial aniso-CA.

**Conclusions:**

Both corneal curvature radius and axial length asymmetry were correlated with aniso-TA. Children with 5-min Apgar score < 7 were more likely to have aniso-TA, while twin or triple, pre-term or post-term delivery were more likely to have vectorial aniso-CA.

**Supplementary Information:**

The online version contains supplementary material available at 10.1186/s12886-021-01808-7.

## Background

Astigmatism occurs when incident light rays do not converge at a single focal point [1]. It can lead to substantial visual dysfunction due to visual torsion, metamorphosis, asthenopia and reduced accommodation response [2, 3]. Some studies suggest astigmatism may also be associated with myopia progression [4, 5]. Anisometropia is an interocular asymmetry in refraction that can be associated with strabismus, amblyopia, aniseikonia, and reduced stereopsis [6–9]. Vision In Preschoolers Study Group (VIP) has demonstrated that non-vectorial aniso-astigmatism was more related with unilateral amblyopia than isometropia [10]. Thus aniso-astigmatism may bring damage to visual development in preschool children.

Previous studies have been focusing on risk factors for astigmatism [11–13]. Maternal smoking during pregnancy, caesarean section, darker iris colour, Hispanic, African American, and Asian race might be risks factor of astigmatism. Few studies explore the risk factors for aniso-astigmatism except the Sydney Myopia Study and the Shandong Children Eye Study [14, 15]. It remains unclear whether maternal factors are associated with aniso-astigmatism in Chinese preschool children. Furthermore, vectorial feature of astigmatism was rarely considered while analyzing the risk factors for aniso-astigmatism [10, 16, 17]. However, initial oblique astigmatism is more likely to be associated with amblyopia than orthogonal astigmatism, even with a small degree [18, 19]. Thus, cylinder axis should not be neglected.

The aim of this study was to describe the characteristics of aniso-TA using cylinder power and by vector analysis, to compare the interocular biometric parameters between with aniso-astigmatism group and without aniso-astigmatism group and to determine risk factors for aniso-astigmatism including vectorial aniso-total astigmatism (vectorial aniso-TA), non-vectorial aniso-TA, vectorial aniso-corneal astigmatism (vectorial aniso-CA) and vectorial aniso-residual astigmatism (vectorial aniso-RA) in a population-based Nanjing Eye Study (NES).

## Methods

### Study design

The NES was designed to prospectively observe the onset and progression of childhood ocular diseases in eastern China, which is an ongoing population-based open cohort study. All study procedures were approved by the institutional review board in The First Affiliated Hospital with Nanjing Medical University and were conducted according to the tenets of the Declaration of Helsinki. Written consent was obtained from the parents or guardians of all children. This study comprised 61- to 72-month-old children enrolled in kindergartens in the Yuhuatai District of Nanjing City in Eastern China. Data from eye examinations and questionnaire presented in this paper were collected in 2017.

### Ocular examinations and questionnaires

Noncycloplegic autorefraction of both eyes using an autorefractor (Cannon RF10; Canon, Tokyo, Japan), measurement of biometric parameters using the optic low-coherent reflectometer (LenStar LS-900; Haag-Streit AG, Koeniz, Switzerland) and other comprehensive eye examinations were performed among all children. Measurement of autorefraction is performed 3 times as selected in the SET mode. While measuring biometric parameters, three consecutive scans were performed. Scans were operated without pupil dilation, in a dimly lit room according to the manufacturers’ guidelines. If the signal-to-noise ratio (SNR) was less than 2.1, another measurement was taken until reliable readings were achieved from each eye. Biometric parameters refer to central corneal thickness (CCT), corneal radius (CR), anterior chamber depth (ACD), lens thickness (LT) and axial length (AL). A comprehensive questionnaire was distributed to legal guardians of each child. The examining procedures and content of the questionnaire have been described in detail previously [20, 21]. The Apgar score is the most widely used index to report the health status of a newborn [22], which is usually evaluated from the following 5 aspects: appearance (color), pulse (heart rate), grimace response (reflexes), activity (muscle tone) and respiration (breathing rate and effort) at 1, 5 and 10 mins after delivery, with the range being 0–10 [23]. In particular, the 5-min Apgar score is categorized as normal (≥7) and abnormal (< 7) in this study.

### Definition

Definition and calculations of TA, CA and RA were described in previous publications. The vector method modified by Thibos was used to decompose vectorial aniso-astigmatism. The calculations are as follows [24]:

$$ SE=S+C/2 $$

$$ {J}_0=\left(-C/2\right)\times \left(\mathit{\cos}\ 2A\right) $$

$$ {J}_{45}=\left(-C/2\right)\times \left(\mathit{\sin}\ 2A\right) $$

where *SE* is the spherical equivalent, *S* is sphere, *C* is the cylinder in minus format, *A* is the cylinder axis, *J*_*0*_ and *J*_*45*_ are the horizontal or vertical and oblique vectors of the cylinder, respectively.

The difference of TA between both eyes was calculated using cylinder power (non-vectorial aniso-TA was defined as ≥1.00 Dioptre Cylinder [DC] between both eyes) and by vector analysis (vectorial aniso-TA was defined as a difference of ≥0.5 in *J*_*0*_ or *J*_*45*_ between both eyes which is equivalent to 1.00 DC) [16]. Similar definitions applied to aniso-CA and aniso-RA. Aniso-*J*_*0t*_ and aniso-*J*_*45t*_ are *J*_*0*_ and *J*_*45*_ of vectorial aniso-TA. Aniso-*J*_*0c*_ and aniso-*J*_*45c*_ are *J*_*0*_ and *J*_*45*_ of vectorial aniso-CA. Aniso-*J*_*0r*_ and aniso-*J*_*45r*_ are *J*_*0*_ and *J*_*45*_ of vectorial aniso-CA. Group A included children with vectorial aniso-TA, and the others belonged to group B. Group C included children with vectorial aniso-CA, and the others belonged to group D. Group E included children with vectorial aniso-RA, and the others belonged to group F. Group G included children with non-vectorial aniso-TA, and the others belonged to group H.

### Statistical analysis

All statistical analyses were performed using the Statistical Package for the Social Sciences (SPSS V.13.0; IBM, Chicago, IL, USA). Two-sample t-test was employed for comparisons of means and chi-square test was employed for comparison of proportions while comparing the characteristics of children included in the analysis with those excluded due to missing data. Prevalence of aniso-TA was compared between boys and girls and between 61- to 66-month-old children and 67- to 72-month-old children. Spearman correlation coefficient (ρ) was used to evaluate the relationships among the components of aniso-astigmatism. Comparisons of interocular difference in biometric parameters were performed between children with vs. without vectorial aniso-astigmatism (Mann-Whitney U test). Chi-square tests for categorical variables and t-tests for continuous variables were used for detection of potentially associated factors. Variables with a P- value < 0.05 were kept in the multivariate logistic regression models. The forward variable selection was performed to determine statistically significant risk factors for each type of aniso-astigmatism. Odds ratios (OR) and their 95% confidence intervals (95% CI) were calculated. All statistical tests were two-sided and *P* < 0.05 was considered statistically significant.

## Results

### Prevalence of aniso-TA

Among 2300 eligible preschoolers, 1920 (participation rate 83.48%) children were examined. A total of 404 children were uncooperative and no refraction measurements or biometric parameters from right or left eyes were obtained after several attempts. Guardians of 385 children did not complete the questionnaires, leaving 1131 children (58.90% of eligible participants) included in this study.

There were no significant differences in characteristics of children (including age, gender, prevalence rate of aniso-TA) between children included in the analysis and those excluded from analysis due to missing data in questionnaire.

The mean (± SD) age was 66.90 ± 3.38 months and 53.31% of participants were boys. Han nationality children (1117, 98.76%) constituted the majority of the population. The prevalence of TA ≥1.00 DC was 12.56% (95% CI = 10.62 to 14.49%) in right eye and 12.73% (95% CI = 10.79 to 14.68%) in left eye. Table 1 shows the prevalence of aniso-TA stratified by sex and age. The prevalence of non-vectorial aniso-TA was 1.95%, while the prevalence of vectorial aniso-TA was 3.89%. Neither non-vectorial aniso-TA nor vectorial aniso-TA varied with sex or age (all *P* > 0.05). Forty-four children had vectorial aniso-TA. Of them, 26 children had aniso-*J*_*0t*_ ≥ 0.5, 24 children had aniso-*J*_*45t*_ ≥ 0.5, and six children had both. In addition, the prevalence of non-vectorial aniso-TA ≥2.00 DC was 0.18% and none had non-vectorial aniso-TA ≥3.00 DC.

**Table 1:** Prevalence of aniso- total astigmatism stratified by sex and age

Characteristics	N (%)	Vectorial aniso-TA ^**a**^ N (%, 95 CI)	***P***	Non-vectorial aniso-TA ^**b**^ N (%, 95 CI)	***P***
Sex			0.77		0.74
Boys	603 (53.3%)	22 (3.65%, 2.15–5.15)		13 (2.16%, 0.99–3.32)	
Girls	528 (46.7%)	22 (4.17%, 2.46–5.88)		9 (1.70%, 0.60–2.81)	
Age (month)			0.82		0.71
61–66	546 (48.3%)	20 (3.66%, 2.08–5.24)		12 (2.20%, 0.95–3.43)	
67–72	685 (51.7%)	24 (3.50%, 2.12–4.88)		10 (1.71%, 0.66–2.76)	
Total	1131 (100%)	44 (3.89%, 2.76–5.02)		22 (1.95%, 1.14–2.75)	

*N* number, *CI* confidence interval

^a^Vectorial aniso-TA was defined as a difference of ≥0.5 in *J*_*0*_ or *J*_*45*_ between the two eyes

^b^Non-vectoral aniso-TA was defined as the difference of ≥1.0 diopter cylinder in absolute cylinder between the two eyes regardless of axis

### The components of vectorial aniso-astigmatism

There was a statistically significant association between aniso-*J*_*0*t_ and aniso-*J*_*0*c_ (ρ = 0.15, *P* < 0.001), and also between aniso-*J*_*45*t_ and aniso-*J*_*45*c_ (ρ = 0.11, *P* < 0.001). There was a statistically significant association between aniso-*J*_*0*t_ and aniso-*J*_*0*r_ (ρ = 0.22, *P* < 0.001), and also between aniso-*J*_*45*t_ and aniso- *J*_*45*r_ (ρ = 0.11, *P* < 0.001).

### Comparison between groups towards interocular biometric parameters

Table 2 shows comparisons of interocular differences in ocular biometric parameters between groups with vs. without aniso-astigmatism. Absolute value of interocular differences in AL, mean CR, AL/CR, CCT, ACD, LT were calculated. The absolute value of interocular differences in AL, CR and AL/CR, ACD were significantly different between group A and group B (*P* = 0.001, *P* < 0.001, *P* = 0.001, and *P =* 0.01 respectively). The absolute value of interocular differences in CR and AL/CR were significantly different between group C and group D (both *P* < 0.001), which were also significantly different between group E and group F (both *P* < 0.001). The absolute value of interocular differences in AL, CR and AL/CR were significantly different between group G and group H (*P* < 0.001, *P* = 0.001, and *P* < 0.001 respectively).

**Table 2:** Comparisons of interocular differences in ocular biometric parameters between groups with vs. without aniso-astigmatism

	AL (mm)		MCR (mm)		AL/CR		CCT (mm)		ACD (mm)		LT (mm)	
	Mean ± SD	***P***	Mean ± SD	***P***	Mean ± SD	***P***	Mean ± SD	***P***	Mean ± SD	***P***	Mean ± SD	***P***
Vectorial aniso-TA group (N)		0.001		< 0.001		0.001		0.19		0.01		0.09
Group A (44)	0.19 ± 0.24		0.12 ± 0.09		0.05 ± 0.05		9.50 ± 17.17		0.09 ± 0.09		0.10 ± 0.10	
Group B (1087)	0.11 ± 0.14		0.07 ± 0.07		0.03 ± 0.03		7.59 ± 14.27		0.06 ± 0.08		0.07 ± 0.09	
Vectorial aniso-CA group (N)		0.89		< 0.001		< 0.001		0.30		0.79		0.78
Group C (278)	0.11 ± 0.14		0.11 ± 0.09		0.04 ± 0.04		7.54 ± 14.74		0.06 ± 0.07		0.08 ± 0.09	
Group D (853)	0.11 ± 0.14		0.06 ± 0.06		0.03 ± 0.03		7.70 ± 14.29		0.06 ± 0.08		0.07 ± 0.10	
Vectorial aniso-RA group (N)		0.48		< 0.001		< 0.001		0.36		0.42		0.87
Group E (273)	0.12 ± 0.15		0.11 ± 0.09		0.04 ± 0.04		7.95 ± 15.18		0.06 ± 0.07		0.07 ± 0.08	
Group F (858)	0.11 ± 0.14		0.06 ± 0.06		0.03 ± 0.03		7.57 ± 14.14		0.06 ± 0.08		0.08 ± 0.10	
Non-vectorial aniso-TA group (N)		< 0.001		0.001		< 0.001		0.97		0.81		0.68
Group G (22)	0.28 ± 0.30		0.13 ± 0.09		0.06 ± 0.06		8.95 ± 17.20		0.06 ± 0.06		0.07 ± 0.09	
Group H(1109)	0.10 ± 0.14		0.07 ± 0.07		0.03 ± 0.03		7.63 ± 14.34		0.06 ± 0.08		0.07 ± 0.09	

All numbers showed in this table were calculated as absolute values of interocular deviation with a form of mean ± standard deviation

*AL* axial length, *MCR* mean corneal curvature radius, *CCT* central corneal thickness, *ACD* anterior chamber depth, *LT* lens thickness, *SD* standard deviation, *N* number

### Risk factors for aniso-astigmatism

Comparisons for each risk factor between group A and group B were shown in sTable 1. Children in group A were more likely to have abnormal 5 min-Apgar score (*P* < 0.001) and parental astigmatism (*P* = 0.03) than those in group B. In the multivariate analysis, two variables remained significantly associated with vectorial aniso-TA: 5 min-Apgar score and parental astigmatism. Children with 5 min-Apgar score lower than 7 were 6.42 times as likely to have vectorial aniso-TA as children with normal Apgar score (95%CI = 2.63–15.69, *P* < 0.001). Children with parental astigmatism were 2.03 times as likely to have vectorial aniso-TA as children without parental astigmatism (95%CI = 1.09–3.79, *P* = 0.03).

Comparisons for each risk factor between group C and group D were shown in sTable 2. Children in group C were more likely to have older father at child birth (*P* = 0.047), pre-term delivery (*P* = 0.01), more outdoor activity (*P* = 0.03) and being twin or triple (*P* = 0.03) than those in group D. In the multivariate logistic regression analysis, two variables remained significantly associated with vectorial aniso-CA: being twin or triple and term delivery (Table 3). Children being twin or triple were 2.43 times as likely to have vectorial aniso-CA as those being monotocous (95%CI = 1.05–5.60, *P* = 0.04). Pre-term delivery (OR = 2.60, 95%CI = 1.09–6.15, *P* = 0.03) and post-term delivery (OR = 3.61, 95%CI = 1.31–9.96, *P* = 0.01) were more likely to have vectorial aniso-CA than full-term delivery.

**Table 3:** Independent Risk Factors for vectorial aniso-CA from Multivariate Logistic Regression

	Multivariate analysis		
Risk factors	Adjusted OR	95% CI	*P*
Twin or triple (Yes vs No)	2.43	1.05–5.60	0.04
Term delivery			
Full-term	Reference		
Pre-term	2.60	1.09–6.15	0.03
Post-term	3.61	1.31–9.96	0.01

*OR* odds ratio, *CI* confidence interval

Comparisons for each risk factor between group E and group F were shown in sTable 3 and no statistically significant difference was found. Likewise, no statistically significant variable was found to be associated with vectorial aniso-RA in the multivariate logistic regression analysis. Comparisons for each risk factor between group G and group H were shown in sTable 4. Children in group G were more likely to have younger paternal age at child birth, (*P* = 0.049) abnormal 5 min-Apgar score (*P* = 0.03) than those in group H. In the multivariate logistic regression analysis, only 5 min-Apgar score remained significantly associated with non-vectorial aniso-TA. Children with 5 min-Apgar score lower than 7 were 4.99 times as likely to have non-vectorial aniso-TA as children with normal Apgar score (95%CI = 1.41–17.68, *P* = 0.01).

## Discussion

This study describes, for the first time to our knowledge, the prevalence of aniso-TA using both non-vectorial aniso-TA and vectorial aniso-TA in Chinese preschool children. The prevalence of vectorial aniso-TA was twice as common as non-vectorial aniso-TA, which did not vary with sex and age. The prevalence of aniso-TA was much lower than that of TA.

Prevalence of aniso-TA from previous studies on similar age population was shown in Table 4. These studies reported different prevalence rate, which might be due to different ethnicity, age, and whether vectorial analysis was used. We compared the prevalence of non-vectorial aniso-TA with previous studies defined as ≥1.0 DC. The prevalence of non-vectorial aniso-TA in the present study was lower than that found in the Tohono O’odham Native American children, in the Northern Ireland Childhood Errors of Refraction (NICER) study and in the rural area of southwestern Japan [25, 26, 29]. However, it was higher than that in the Sydney Myopia Study, and similar to that in the Sydney Paediatric Eye Disease Study [14, 28]. Among these studies, the prevalence of non-vectorial aniso-TA found in the Tohono O’odham Native American children was the highest, in accordance with the population’s high TA prevalence [27, 29]. When compared with the Shandong Children Eye Study, which was also carried out among Chinese children, the prevalence in this study was lower [15]. Our previous study also showed the TA prevalence was lower than that from The Shandong Children Eye Study [20, 30]. Few studies revealed the prevalence of vectorial aniso-TA. The prevalence of vectorial aniso-TA in the present study was lower than that found in the Multi-Ethnic Pediatric Eye Disease Study (MEPEDS) [16]. In their study, vectorial aniso-TA was twice as common as non-vectorial aniso-TA, similar with our results. Children in MEPEDS were African American and Hispanic, who also showed higher TA prevalence. The difference between non-vectorial aniso-TA prevalence and vectorial aniso-TA prevalence was reasonable as vectorial aniso-TA took astigmatic axis into consideration.

**Table 4:** Studies of aniso-astigmatism among young children

Author	Year	Location	Age	Sample size	Definition	Prevalence
The Sydney Myopia Study [14]	2006	Sydney, Australia	6 years	1765	≥ 1.0 DC	1.60%
Dobson et al. [25]	2008	Tohono O’odham, American	4–13 years	1041	≥ 1.0 DC	15%
The Northern Ireland Childhood Errors of Refraction Study [26]	2013	Northern Ireland, England	6–7 years	661	≥ 1.0 DC	7.70%
			12–13 years	389		5.60%
The Sydney Paediatric Eye Disease Study [28]	2013	Sydney, Australia	6–72 months	2090	≥ 1.0 DC	Overall 3%
						European-Caucasian 1.9%
						East-Asian 5.2%
						South-Asian 3.6%
						Middle-Eastern 3.3%
The Multi-Ethnic Pediatric Eye Disease Study [16]	2010	California, America	6–72 months	Hispanic American, 3030	≥ 1.0 DC	5.60%
					≥ 0.50 in *J*_*0*_ / *J*_45_	10.40%
				African American, 2994	≥ 1.0 DC	4.50%
					≥ 0.50 in *J*_*0*_ / *J*_45_	11.90%
The Shandong Children Eye Study [15]	2015	Shandong, China	4–18 years	6025	≥ 1.0 DC	3.70%
Yamashita et al. [27]	1997	Rural area of southwestern Japan	6 years	350	≥ 1.0 DC	2.6%
			7 years			2.3%
			8 years			2.0%
			9 years			3.4%
			10 years			3.7%
			11 years			4.3%

Whatever definition was used, aniso-TA was associated not only with increased interocular differences in CR, but also with AL, possibly due to the relationship among aniso-TA and anisometropia. A similar correlation was reported by Huynh et al. [14], O’Donoghue et al. [25], Singh et al. [31], and Hameshi et al. [32]. These studies showed non-vectorial aniso-CA was associated with non-vectorial aniso-TA. This finding is in agreement with our knowledge that most aniso-TA of the eyes is due to corneal issues. Interestingly, we found that interocular differences in ACD were associated with vectorial aniso-TA. The finding is in accordance with Hameshi et al., but contradicts with the results of the NICER Study [25, 32]. Vectorial aniso-CA and vectorial aniso-RA can only be explained by interocular differences in CR.

This study revealed that parental astigmatism was a risk factor for vectorial aniso-TA. Our previous article has presented the contradictory results from different studies on the genetic contribution to astigmatism [21]. Similar condition exists on the genetic contribution to aniso-astigmatism. Recently, a population-based twin study showed that the correlation between monozygotic twins for aniso-CA were significantly different from dizygotic twins [33]. A study in Korea found that intraclass correlation coefficients for spherical equivalent and ocular biometrics were significantly higher in monozygotic twins compared with singleton, with greater consistency and conformity [34]. However, another study did not find any significant difference between children being twin or siblings in refractive error, corneal curvature, ACD and CCT [35]. Further investigations may be needed to clarify the relationship between genetics and aniso-astigmatism.

Our study showed that children with a 5-min Apgar score < 7 had a higher likelihood of developing aniso-TA at 5- to 6- years compared to those with an Apgar score of 7–10 (within the normal range), while pre-term or post-term delivery were risk factors for vectorial aniso-CA. A previous study found asymmetrical growth restriction in perterm-born children [36]. Dubois reported structural asymmetries of perisylvian regions in the preterm newborn [37]. Additionally, several studies have found abnormal nervous system function in preterm born children. Michalczuk suggested that Apgar score seemed to be a predicting factor for developmental rate of brain function in children with history of prematurity [38]. Teli found that low 5-min Apgar score in very perterm infants hindered corpus callosum microstructural development [39]. Moreover, eye growth is parellel to neurodevelopment. White matter changes were found in children with anisometropic amblyopia [40]. It has also been reported that low 5-min Apgar score increased the risk of reduced vision in children [41]. The Sydney Myopia Study found that paternal age > 35 years was accociated with non-vectorial aniso-TA in unadjusted analyses. After multivariable adjustment, breast feeding had a significant protective association (*P* = 0.02) with non-vectorial aniso-TA. In our study, neither paternal age > 35 years or breast feeding was a risk factor for non-vectorial aniso-TA. To sum up, intrauterine hypoplasia and poor birth condition may be associated with asymmeric whole body development, neurodevelopment, and asymmeric visual and refractive development such as aniso-astigmatism. Further work is required to clarify the developmental mechanism behind these associations.

Astigmatism is relatively complicated because of its vectorial feature. Both non-vectorial analysis and vectorial analysis were included in this study. The former only considers the cylinder power of aniso-astigmatism and neglects its axis. The latter, used frequently now to decompose aniso-astigmatism, also has its limitation as it splits aniso-astigmatism to two directions. However, how to integrate *J*_*0*_ and *J*_*45*_ difference is still a problem. Both methods describing aniso-astigmatism have their shortcomings but we hope to improve understanding and promote exploration of aniso-astigmatism using these two methods together.

The strengths of this study include its population-based design, large sample size, and standardized examination protocols performed by an expert team, risk factors during pregnancy and early childhood. Our analyses are different from most previous studies by considering vectorial features of aniso-astigmatism. The limitation of this study is that some eligible children were not included into the anaysis due to missing data in questionnaire or refractive error measures, the risk factor data collected through questionnaire may be subjective and biased. What’ more, this study is also limited in the use of refraction data under noncyloplegic condition. Autorefractors have been widely used in vision screening, clinical practice, and researches, especially in epidemiological surveys and clinical trials, to check the refractive status of children. Fogging mechanisms were usually built-in to reduce influence of accommodation, whereas the influence could not be fully eliminated. Several studies have demonstrated that noncycloplegic autorefraction has reasonable accuracy and repeatability compared with cycloplegic retinoscopy [42–44]. A previous study measures refraction before and after cycloplegia using the autorefractor Canon RK-F1. Results showed statistically significant differences between the cycloplegic and noncycloplegic spherical powers, but insignificant differences between the cycloplegic and noncycloplegic cylindrical values [42]. A study among Chinese children found significant differences between cycloplegic and non-cycloplegic spherical euivalent using Canon RK-F1, but insignificant differences between cycloplegic and non-cycloplegic *J*_*0*_ and *J*_*45*_ [43]. Another recent study showed that measurements obtained with the closed-field autorefractor Topcon KR-800 without cycloplegia had good reliability for the evaluation of spherical equivalent and *J*_*0*_, indicating that it has reliable accuracy of measurement of the “with-the-rule” and “against-the-rule” astigmatism, whereas agreement with cycloplegic retinoscopy for the evaluation of *J*_*45*_ was fair to good, indicating potential limitations for the detection of oblique astigmatism [44]. Cycloplegia may bring vision discomfort, inconvenience, and allergic reaction and takes time. Thus it’s less accepted by children and guardians. Actually, it’s hard to achieve cycloplegic measurements at high proportion among preschool children. We admit that noncycloplegic autorefraction has its shortage but still we think it can provide referable measurements for cylindrical power. We didn’t include noncycloplegic spherical data considering the relatively more significant influence of cycloplegia on spherical euivalent. It has been reported that for children, when cycloplegic refraction is difficult to perform, AL/CR may be the second choice in predicting spherical equivalent [45, 46]. Therefore we include AL/CR in the analyses instead.

## Conclusions

In summary, in the 61- to 72-month-old children in the Yuhuatai District, the prevalence of non-vectorial aniso-TA was 1.95%, while the prevalence of vectorial aniso-TA was twice as common as non-vectorial aniso-TA. Both CR and AL asymmetry were correlated with aniso-TA. Children with 5-min Apgar score < 7 were more likely to have aniso-TA, while twin or triple, pre-term or post-term delivery were more likely to have vectorial aniso-CA.

## Supplementary Information

**Additional file 1: Table S1.** Distribution of Risk Factors in Children With vs. Without Vectorial Aniso-Total Astigmatism.

**Additional file 2: Table S2.** Distribution of Risk Factors in Children With vs. Without Vectorial Aniso-Corneal Astigmatism.

**Additional file 3: Table S3.** Distribution of Risk Factors in Children With vs. Without Vectorial Aniso-Residual Astigmatism.

**Additional file 4: Table S4.** Distribution of Risk Factors in Children With vs. Without Non-Vectorial Aniso-Total Astigmatism.

## Data Availability

Data can be shared upon request.

## Abbreviations

TA: Total Astigmatism
CA: Corneal Astigmatism
RA: Resiual Astigmatism
DC: Dioptre Cylinder
CCT: Central Corneal Thickness
CR: Corneal Radius
ACD: Anterior Chamber Depth
LT: Lens Thickness
AL: Axial Length
