# Supplementary material for: The biometric parameters of aniso-astigmatism and its risk factor in Chinese preschool children: the Nanjing eye study
Source: BMC Ophthalmol. 2021 Feb 3;21:67. doi: 10.1186/s12886-021-01808-7 (PMC7860027; doi:10.1186/s12886-021-01808-7)
Supplement: Supplementary file 1 — Additional file 1: Table S1. Distribution of Risk Factors in Children With vs. Without Vectorial Aniso-Total Astigmatism. [file 12886_2021_1808_MOESM1_ESM.docx]

**sTable 1. Distribution of Risk Factors in Children With vs. Without Vectorial Aniso-Total Astigmatism**

| **Risk Factors** | **Group A (N=44)** | **Group B (N=1087)** | ***P*-value** |
| --- | --- | --- | --- |
| Mean (± SD) age (month) | 67.07 ± 2.99 | 66.89 ± 3.40 | 0.053 |
| Gender: male (%) | 22 (50%) | 581 (53.45%) | 0.77 |
| Mean (± SD) paternal age at child birth (year) | 26.91 ± 3.78 | 27.83 ± 4.84 | 0.24 |
| Mean (± SD) maternal age at child birth (year) | 25.75 ± 3.13 | 26.15 ± 3.96 | 0.21 |
| Paternal myopia: yes (%) | 16 (36.36%) | 389 (35.79%) | 0.94 |
| Maternal myopia: yes (%) | 23(52.27%) | 426(39.19%) | 0.09 |
| Parental astigmatism: yes (%) | 18 (40.91%) | 270 (24.84%) | **0.03** |
| Mode of pregnancy: assisted (%) | 5 (11.36%) | 182 (16.74%) | 0.46 |
| Term delivery |  |  | 0.07 |
| Full-term | 40 (90.91%) | 979 (90.06%) |  |
| Pre-term | 4 (9.09%) | 55 (5.06%) |  |
| Post-term | 0 (0%) | 53 (4.88%) |  |
| Mean (± SD) birth weight (kilogram) | 3.19 ± 0.53 | 3.35 ± 0.52 | 0.76 |
| 5-min Apgar score: abnormal (%) | 7 (15.91%) | 30 (2.76%) | **<0.001** |
| Delivery mode |  |  | 0.96 |
| Vaginal | 25 (56.82%) | 610 (56.12%) |  |
| Vaginal transferring to cesarean | 3 (6.82%) | 87 (8.00%) |  |
| Cesarean | 16 (36.36%) | 390 (35.88%) |  |
| Oxygen uptake after birth: yes (%) | 6 (13.64%) | 63 (5.80%) | 0.07 |
| Second or third child: yes (%) | 6 (13.64%) | 213 (19.60%) | 0.43 |
| Twin or triple: yes (%) | 1 (2.27%) | 23 (2.12%) | 0.94 |
| Feeding patterns |  |  | 0.36 |
| Exclusive breastfeeding | 19 (43.18%) | 525 (48.30%) |  |
| Partial breastfeeding | 18 (40.91%) | 464 (42.69%) |  |
| Formula feeding | 7 (15.91%) | 98 (9.01%) |  |
| Second-hand smoke exposure during pregnancy: yes (%) | 9 (20.45%) | 148 (13.62%) | 0.29 |
| Maternal working during pregnancy: yes (%) | 19 (43.18%) | 505 (46.46%) | 0.79 |
| Mean (± SD) outdoor activity (hour) | 2.03 ± 0.78 | 2.25 ± 1.42 | 0.054 |
| Mean (± SD) mid-working distance activity (hour) | 4.69 ± 2.14 | 4.79 ± 3.53 | 0.59 |
| Mean (± SD) near-work activity (hour) | 1.21 ± 0.89 | 1.55 ± 1.68 | 0.25 |

Group A: children with vectorial aniso-total astigmatism; Group B: children with vectorial aniso-total astigmatism
