# Supplementary material for: The biometric parameters of aniso-astigmatism and its risk factor in Chinese preschool children: the Nanjing eye study
Source: BMC Ophthalmol. 2021 Feb 3;21:67. doi: 10.1186/s12886-021-01808-7 (PMC7860027; doi:10.1186/s12886-021-01808-7)
Supplement: Supplementary file 2 — Additional file 2: Table S2. Distribution of Risk Factors in Children With vs. Without Vectorial Aniso-Corneal Astigmatism. [file 12886_2021_1808_MOESM2_ESM.docx]

**sTable 2. Distribution of Risk Factors in Children With vs. Without Vectorial Aniso-Corneal Astigmatism**

| **Risk Factors** | **Group C (N=278)** | **Group D (N=873)** | ***P*-value** |
| --- | --- | --- | --- |
| Mean (± SD) age (month) | 66.85 ± 3.38 | 66.91 ± 3.39 | 0.98 |
| Gender: male (%) | 135 (48.56%) | 468 (42.20%) | 0.08 |
| Mean (± SD) paternal age at child birth (year) | 28.00 ± 4.40 | 27.73 ± 4.93 | **0.047** |
| Mean (± SD) maternal age at child birth (year) | 26.20 ± 3.55 | 26.12 ± 4.05 | 0.15 |
| Paternal myopia: yes (%) | 99 (35.61%) | 306 (35.87%) | 0.99 |
| Maternal myopia: yes (%) | 108 (38.85%) | 341 (39.98%) | 0.79 |
| Parental astigmatism: yes (%) | 76 (27.34%) | 212 (24.85%) | 0.46 |
| Mode of pregnancy: assisted (%) | 53 (19.06%) | 134 (15.71%) | 0.22 |
| Term delivery |  |  | **0.01** |
| Full-term | 252 (90.65%) | 767 (89.92%) |  |
| Pre-term | 20 (7.19%) | 39 (4.57%) |  |
| Post-term | 6 (2.16%) | 47 (5.51%) |  |
| Mean (± SD) birth weight (kilogram) | 3.29 ± 0.56 | 3.36 ± 0.50 | 0.33 |
| 5-min Apgar score: abnormal (%) | 13 (4.68%) | 24 (2.81%) | 0.19 |
| Delivery mode |  |  | 0.39 |
| Vaginal | 157 (56.47%) | 478 (56.04%) |  |
| Vaginal transferring to cesarean | 17 (6.12%) | 73 (8.56%) |  |
| Cesarean | 104 (37.41%) | 302 (35.40) |  |
| Oxygen uptake after birth: yes (%) | 22 (7.91%) | 47 (5.51%) | 0.19 |
| Second or third child: yes (%) | 52 (18.70%) | 167 (19.58%) | 0.82 |
| Twin or triple: yes (%) | 11 (3.96%) | 13 (1.52%) | **0.03** |
| Feeding patterns |  |  | 0.28 |
| Exclusive breastfeeding | 124 (44.60%) | 420 (49.24%) |  |
| Partial breastfeeding | 130 (46.76%) | 352 (41.27%) |  |
| Formula feeding | 24 (8.63%) | 81 (9.50%) |  |
| Second-hand smoke exposure during pregnancy: yes (%) | 39 (14.03%) | 118 (13.83%) | 0.94 |
| Maternal working during pregnancy: yes (%) | 129 (46.40%) | 395 (46.31%) | 0.98 |
| Mean (± SD) outdoor activity (hour) | 2.32 ± 1.71 | 2.21 ± 1.28 | **0.03** |
| Mean (± SD) mid-working distance activity (hour) | 4.97 ± 4.10 | 4.73 ± 3.25 | 0.88 |
| Mean (± SD) near-work activity (hour) | 1.48 ± 1.83 | 1.55 ± 1.60 | 0.98 |

Group C: children with vectorial aniso-corneal astigmatism; Group D: children with vectorial aniso-corneal astigmatism
