# Supplementary material for: The biometric parameters of aniso-astigmatism and its risk factor in Chinese preschool children: the Nanjing eye study
Source: BMC Ophthalmol. 2021 Feb 3;21:67. doi: 10.1186/s12886-021-01808-7 (PMC7860027; doi:10.1186/s12886-021-01808-7)
Supplement: Supplementary file 3 — Additional file 3: Table S3. Distribution of Risk Factors in Children With vs. Without Vectorial Aniso-Residual Astigmatism. [file 12886_2021_1808_MOESM3_ESM.docx]

**sTable 3. Distribution of Risk Factors in Children With vs. Without Vectorial Aniso-Residual Astigmatism.**

| **Risk Factors** | **Group E (N=273)** | **Group F (N=858)** | ***P*-value** |
| --- | --- | --- | --- |
| Mean (± SD) age (month) | 66.73 ± 3.37 | 66.95 ± 3.39 | 0.74 |
| Gender: male (%) | 140 (51.28%) | 463 (53.96%) | 0.48 |
| Mean (± SD) paternal age at child birth (year) | 27.79 ± 4.58 | 27.80 ± 4.87 | 0.45 |
| Mean (± SD) maternal age at child birth (year) | 26.13 ± 3.68 | 26.15 ± 4.00 | 0.50 |
| Paternal myopia: yes (%) | 94 (34.43%) | 311 (36.25%) | 0.64 |
| Maternal myopia: yes (%) | 107 (39.19%) | 342 (39.86%) | 0.90 |
| Parental astigmatism: yes (%) | 74 (27.11%) | 214 (24.94%) | 0.53 |
| Mode of pregnancy: assisted (%) | 49 (17.95%) | 138 (16.08%) | 0.53 |
| Term delivery |  |  | 0.19 |
| Full-term | 248 (90.84%) | 771 (89.86%) |  |
| Pre-term | 17 (6.23%) | 42 (4.90%) |  |
| Post-term | 8 (2.93%) | 45 (5.24%) |  |
| Mean (± SD) birth weight (kilogram) | 3.33 ± 0.53 | 3.34 ± 0.51 | 0.83 |
| 5-min Apgar score: abnormal (%) | 12 (4.40%) | 25 (2.91%) | 0.32 |
| Delivery mode |  |  | 0.58 |
| Vaginal | 146 (53.48%) | 489 (56.99%) |  |
| Vaginal transferring to cesarean | 24 (8.79%) | 66 (7.70%) |  |
| Cesarean | 103 (37.73%) | 303 (35.31%) |  |
| Oxygen uptake after birth: yes (%) | 20 (7.33%) | 49 (5.71%) | 0.41 |
| Second or third child: yes (%) | 49 (17.95%) | 170 (19.81%) | 0.55 |
| Twin or triple: yes (%) | 6 (2.20%) | 18 (2.10%) | 0.92 |
| Feeding patterns |  |  | 0.69 |
| Exclusive breastfeeding | 131 (47.99%) | 413 (48.14%) |  |
| Partial breastfeeding | 120 (43.96%) | 120 (13.99%) |  |
| Formula feeding | 22 (8.06%) | 22 (2.56%) |  |
| Second-hand smoke exposure during pregnancy: yes (%) | 40 (14.65%) | 117 (13.64%) | 0.75 |
| Maternal working during pregnancy: yes (%) | 131 (47.99%) | 393 (45.80%) | 0.58 |
| Mean (± SD) outdoor activity (hour) | 2.22 ± 1.39 | 2.25 ± 1.40 | 0.94 |
| Mean (± SD) mid-working distance activity (hour) | 4.96 ± 4.51 | 4.73 ± 3.09 | 0.27 |
| Mean (± SD) near-work activity (hour) | 1.60 ± 2.30 | 1.51 ± 1.40 | 0.11 |

Group E: children with vectorial aniso-residual astigmatism; Group F: children with vectorial aniso-residual astigmatism
