# Supplementary material for: The biometric parameters of aniso-astigmatism and its risk factor in Chinese preschool children: the Nanjing eye study
Source: BMC Ophthalmol. 2021 Feb 3;21:67. doi: 10.1186/s12886-021-01808-7 (PMC7860027; doi:10.1186/s12886-021-01808-7)
Supplement: Supplementary file 4 — Additional file 4: Table S4. Distribution of Risk Factors in Children With vs. Without Non-Vectorial Aniso-Total Astigmatism. [file 12886_2021_1808_MOESM4_ESM.docx]

**sTable 4. Distribution of Risk Factors in Children With vs. Without Non-Vectorial Aniso-Total Astigmatism**

| **Risk Factors** | **Group G (N=22)** | **Group H (N=1109)** | ***P*-value** |
| --- | --- | --- | --- |
| Mean (± SD) age (month) | 66.50 ± 2.92 | 66.91 ± 3.40 | 0.15 |
| Gender: male (%) | 13 (59.09%) | 590 (53.20) | 0.74 |
| Mean (± SD) paternal age at child birth (year) | 26.91 ± 2.72 | 27.82 ± 4.83 | 0.049 |
| Mean (± SD) maternal age at child birth (year) | 25.77 ± 3.10 | 26.15 ± 3.95 | 0.51 |
| Paternal myopia: yes (%) | 10 (45.45%) | 395 (35.62%) | 0.47 |
| Maternal myopia: yes (%) | 11 (50.00%) | 438 (39.50%) | 0.44 |
| Parental astigmatism: yes (%) | 9 (40.91%) | 279 (25.16%) | 0.15 |
| Mode of pregnancy: assisted (%) | 7 (31.82%) | 180 (16.23%) | 0.10 |
| Term delivery |  |  | 0.99 |
| Full-term | 20 (90.90%) | 999 (90.08%) |  |
| Pre-term | 1 (4.55%) | 58 (5.23%) |  |
| Post-term | 1 (4.55%) | 52 (4.69%) |  |
| Mean (± SD) birth weight (kilogram) | 3.39 ± 0.43 | 3.34 ± 0.52 | 0.34 |
| 5-min Apgar score: abnormal (%) | 3 (13.64%) | 34 (3.07%) | **0.03** |
| Delivery mode |  |  | 0.16 |
| Vaginal | 8 (36.36%) | 627 (56.54%) |  |
| Vaginal transferring to cesarean | 3 (13.64%) | 87 (7.84%) |  |
| Cesarean | 11 (50.00%) | 395 (35.62%) |  |
| Oxygen uptake after birth: yes (%) | 3 (13.64%) | 66 (5.95%) | 0.30 |
| Second or third child: yes (%) | 2 (9.09%) | 217 (19.57%) | 0.33 |
| Twin or triple: yes (%) | 1 (4.55%) | 23 (2.07%) | 0.96 |
| Feeding patterns |  |  | 0.33 |
| Exclusive breastfeeding | 8 (36.36%) | 536 (48.33%) |  |
| Partial breastfeeding | 10 (45.46%) | 472 (42.56%) |  |
| Formula feeding | 4 (18.18%) | 101 (9.11%) |  |
| Second-hand smoke exposure during pregnancy: yes (%) | 2 (9.09%) | 155 (13.98%) | 0.73 |
| Maternal working during pregnancy: yes (%) | 11 (50.00%) | 513 (46.26%) | 0.89 |
| Mean (± SD) outdoor activity (hour) | 1.82 ± 0.63 | 2.25 ± 1.41 | 0.07 |
| Mean (± SD) mid-working distance activity (hour) | 4.47 ± 1.90 | 4.80 ± 3.51 | 0.35 |
| Mean (± SD) near-work activity (hour) | 1.29 ± 0.85 | 1.54 ± 1.67 | 0.32 |

Group G: children with non-vectorial aniso-total astigmatism; Group H: children with non-vectorial aniso-total astigmatism
